# Cytotoxicity of unsaturated fatty acids in fresh human tumor explants: concentration thresholds and implications for clinical efficacy

**DOI:** 10.1186/1476-511X-8-54

**Published:** 2009-12-15

**Authors:** David E Scheim

**Affiliations:** 1 3300 Old Farm Road, Blacksburg, VA 24060, USA

## Abstract

**Background:**

Unsaturated fatty acids (UFAs) exhibit *in vitro *cytotoxicity against many malignant cell lines and yield decreased cancer incidence and reduced tumor growth in animal models. But clinical and animal studies to date have achieved response using only localized delivery methods such as intratumoral infusion. To explore possibilities for enhanced clinical efficacy, fresh surgical explants of tumors from 22 patients with five malignancies were exposed to γ-linolenic acid (GLA) and α-linolenic acid (ALA) and analyzed with an *in vitro *chemosensitivity testing system, the Fluorescent Cytoprint Assay (FCA). A total of 282 micro-organ cultures derived from these malignant tumors were exposed to GLA and ALA at different concentrations.

**Results:**

GLA and ALA exhibited greater than 90% cytotoxicity at a sharp concentration threshold between 500 μM and 1 mM against all but two malignant micro-organ cultures tested in 5-10% serum. In tests using 30-40% serum, GLA and ALA killed tumor at concentrations of 2 mM and above.

**Conclusions:**

The concentration threshold of 500 μM to 2 mM exhibited for antitumor activity by GLA and ALA is much higher than that observed in most previously reported cell culture studies but consistent with physiological concentrations found to kill tumor clinically and in animals. A mechanism of antitumor activity by unsaturated fatty acids through selective destabilization of the malignant plasma membrane is considered. An oral regimen is proposed for phase I clinical testing that could push the area under the curve for serum concentration of unbound unsaturated fatty acids over time to much higher levels than previously achieved for systemic administration and into the range that could yield antitumor response.

## Background

Unsaturated fatty acids (UFAs), including the ω-6 agent GLA and the ω-3 agent ALA, have been observed to exhibit cytotoxicity [1-8] or growth inhibition [9-15] against a variety of malignant cell lines. These effects, studied closely over the past two decades, are usually selective for malignant versus normal cells [1-3,5,10,12,16-18]. Concentrations of different UFAs used in these *in vitro *studies were typically no greater than 100 μM, with close to 100% cytotoxicity achieved at concentrations of, for example, 10-12 μM [2,5], 50 μM [1] or 70 μM [19].

At low levels, ω-6 fatty acids have been shown to promote tumor growth in some *in vitro *[15,20,21] and animal [22,23] studies. Retrospective human studies in which tissue composition was analyzed have shown inverse correlations of cancer incidence and ratios of ω-3 to ω-6 fatty acids [24,25]. Benefits of ω-3 supplementation have been demonstrated prospectively in a double-blind study of 27 patients with stage 1 or 2 colon carcinoma or adenomatous polyps randomized to consume 9 grams daily of either fish oil with high eicosapentaenoic acid content or corn oil after surgical excision of detectable lesions. S-phase BrdUrd labeling of tissue from proctoscopic mucosal biopsies, a predictor for incidence of new neoplasms, dropped to 29% of its baseline value after six months for the ω-3 supplemented group but rose in the corn oil-supplemented group [26].

At higher physiological concentrations of GLA achieved through intratumoral administration in subcutaneous pancreatic tumors in nude mice [27] and in rat gliomas [22,28], significant tumor shrinkage was observed. Clinically, major response was reported for 15 glioma patients who after surgery had 1 mg of GLA injected into residual tumor daily via a cerebral catheter over 10 consecutive days. Striking changes in CT scans indicative of response were reported for all patients, and 28 months after study completion, 12 surviving patients were reported under active follow-up with no new symptoms [29]. Partial responses of 40-60% for six glioma patients were reported in a similar prior study [30]. A subsequent study using the same 1 mg daily intratumoral GLA dosage over only 7 days, however, yielded lesser partial responses in 9 patients with grade 4 glioma and no survival gains [31,32]. None of these studies were controlled clinical trials.

Response was also achieved in a phase I clinical trial of intra-vesicle treatment of superficial bladder cancer with Meglumine GLA at concentrations of 2.4 mM or 6 mM for a one-hour retention period. Using these high GLA concentrations, complete responses for 13% and partial responses for 30% of 30 total patients were achieved [33]. But a phase III clinical trial of oral or intravenous lithium GLA at daily doses of less than 1 g/kg for advanced pancreatic cancer yielded only marginal benefits [34]. Clinical studies of low-dose oral GLA for colon [35] and liver [36] cancer likewise yielded no benefits. These successful results for high levels of GLA using localized treatment approaches in contrast to negative outcomes with low levels of GLA obtained using intravenous or oral systemic administration routes are consistent with *in vitro *[6,7,14,15,37] and animal [22,38] studies indicating that UFA antitumor activity is highly dose-dependent.

A particularly sharp dose-response effect for UFA agents was demonstrated with 16 rats treated with GLA eight days after surgical implantation of C6 glioma tumors [22]. GLA was administered via intratumoral infusion at a flow rate of 1 μl/h over a period of 3 or 7 days, with tumor mass assessed the day after treatment completion. In tumors infused with 2 mM GLA administered over 7 days, tumor area was less than 50% of the control subgroup with much of the tumor tissue necrotic but with intact histology in bordering normal brain tissue. Significant tumor growth in comparison to controls, however, was exhibited for the 1 mM, 40 μM and 20 μM GLA concentration subgroups. When GLA was administered intratumorally over 3 days, tumor kill was likewise observed but to a lesser degree at a 2 mM concentration, with growth promotion observed at 1 mM.

To model clinical dose-response effects and explore possibilities for enhanced clinical efficacy, fresh surgical explants of malignant tumors from 22 patients were exposed to unsaturated fatty acids at different concentrations and analyzed using the Fluorescent Cytoprint Assay (FCA). These tumors were pathologically identified as ovarian carcinoma for 18 of the patients and as breast carcinoma, pancreatic carcinoma, melanoma, and glioblastoma, one each, for the other four. Developed in 1986 by Boris Rotman, the FCA assesses the viability of three-dimensional micro-organs derived from fresh surgical human tumor explants following exposure typically to standard chemotherapeutic agents [39,40]. This technique [41-43] and other *in vitro *testing systems [44,45] have been used in clinical practice for chemosensitivity testing since the mid-1980s. The FCA is an attractive experimental model as it provides analytical capabilities of standard *in vitro *systems, allowing about 50 or more viability tests per specimen depending upon size of surgical explant, yet more closely simulates the physiological environment based upon its fresh human tissue source and three-dimensional tumor architecture.

The FCA was found 90% accurate in predicting chemotherapeutic treatment outcomes in a collaborative retrospective study [42]. Clinical response achieved for most agents was at peak plasma levels close to concentrations tested with the FCA procedure [42]. Although the FCA experiments of this present study were not designed to amass a sufficiently large set of well-structured data to support statistical analysis, the basic dose-response characteristics of UFAs that were examined are of particular interest in light of findings indicating potential clinical application [22,32,33] that have emerged since these experiments were conducted.

## Methods

### Surgical explants

Freshly excised human tumor explants were obtained from surgeries through the National Disease Research Interchange (NDRI) and shipped overnight in cold culture medium. Additional specimens were obtained from medical centers in Providence, Rhode Island associated with the Brown University School of Medicine. Normal stromal tissues excised in the vicinity of some tumors were also studied. Malignant tumor specimens from 22 patients and a benign ovarian tumor specimen from an additional patient were received and assayed between March 1994 and March 1996. Of the 22 malignant tumors, 18 were pathologically identified as primary or metastatic ovarian adenocarcinoma and the others identified as breast carcinoma, pancreatic carcinoma, melanoma, and glioblastoma (one of each). For one patient, both primary and metastatic ovarian adenocarcinoma was obtained and studied.

### Materials

Culture chambers were constructed and fluorescein monoacetate synthesized and purified as previously described [39,40]. Culture medium was RPMI 1640 supplemented with 5-40% fetal bovine serum (FBS) or human serum and penicillin-streptomycin (10 U/mL) (GIBCO, Grand Island, NY). Stock collagen solutions were made by diluting a purified collagen (Collagen Corp., Palo Alto., CA) solution (2 mg/ml) with 7.5 times concentrated solution of Dulbecco's modified Eagle powder medium (GIBCO), FBS, and culture medium in the proportion 3.0: 0.5: 0.25: 1.0, respectively. The final solution was used as stock and kept at 2°C. Fresh collagenase (Sigma, St. Louis, MO) was prepared by dissolving the enzyme (300-350 U/mg) in culture medium and sterilized by passage through a 0.22 μm Vanex filter unit (Vanguard International, Neptune, NJ).

### FCA assay procedure

Processing was begun 2-5 hours after receipt of each surgical explant per the methodology previously described [39,40]. Briefly, the tissue was fragmented by first mincing it with scalpels into pieces of about 1 mm^3 ^and then processed for a short period (2-5 seconds) in a Tissumizer (Tekmar). After additional processing and vital staining with fluorescein monoacetate, tumor tissue yielded discrete cellular aggregates, termed "micro-organs," that were easily visualized and manually collected using a Pasteur pipette. These micro-organs correspond to discrete clusters of malignant cells characteristically seen in histological preparations, most distinctly observed in carcinomas [39]. In contrast, normal stromal tissue, not previously studied in the FCA assay, did not naturally aggregate into micro-organs and required additional shearing to separate into small cellular aggregates of equivalent size (which are also termed "micro-organs" in the discussion below). From a typical tissue specimen, 500-700 micro-organs were obtained, each containing 100-2000 viable cells.

Viable micro-organs were then plated as previously described [39,40], 4-40 per micro-organ culture, with a typical yield of 55-90 micro-organ cultures per culture matrix. The matrix was placed on a metal grid and partially submerged into growth medium and incubated at 37° under 5% CO_2 _for periods up to 10 days. In all experiments, cultures without added test agents, typically at least four, were used as controls to assess baseline viability over the course of the experiment. All experiments were performed in the laboratory of Boris Rotman at Brown University, Providence, Rhode Island, where the FCA assay system was used for preclinical screening of novel anticancer agents, with specifications for agents used in these experiments provided by the author.

Through transient, non-toxic staining by fluorescein monoacetate, a digital image of each individual micro-organ culture, termed a "fluorescent cytoprint," was used to assess cytotoxicity of added agents. Fluorescent cytoprints were obtained for each micro-organ culture before and after exposure to agents, and the fluorescence change was quantitatively assessed and used as an index of viability. Greater than 90% viability was scored "resistant;" less than 10% viability was scored "sensitive."

Values in between were scored "intermediate," and borderline values such as e.g. 85% viability were scored intermediate-resistant and 15% viability scored intermediate-sensitive. Because such intermediate values between 10% and 90% viability (which comprised only 14% of all reported viability results) could represent delayed cytotoxic effect, probabilistic variations, or tumor heterogeneity [39], these are less reliable indicators of the activity of agents tested. For the purposes of charting results in the figures shown, characterizations of sensitive, intermediate-sensitive, intermediate, intermediate-resistant, and resistant were assigned numeric values, respectively, of 0, 0.25., 0.5, 0.75 and 1.0.

Readings were performed 5-9 days after the introduction of agents; a 7-day period was used in all but three experiments. In some experiments, additional readings were taken between 3 and 9 days after introduction of agents. Micro-organ cultures were typically exposed to agents until the time of final FCA fluorescence readings. In 33 micro-organ cultures from three malignant specimens, agents were replaced with culture media after a 2-day period, and in 36 micro-organ cultures from two malignant specimens, agents were replaced with culture media after a 5-day period.

### UFAs and other agents used

In the first four experiments, GLA and ALA were studied either as lithium salts or as free fatty acids dissolved 40 mg/ml in ethanol and then added to culture media in a stable suspension to achieve desired agent concentrations, per a methodology used in some prior *in vitro *studies [17,46]. In subsequent experiments, all tests were performed using the sodium salts of GLA and ALA to preclude any possible active effects of ethanol or lithium (no differences in results for the different forms of these agents were in fact observed). Linoleic acid (LA), eicosapentaenoic acid (EPA), and oleic acid (OA) were also tested using their sodium salts. LA and EPA were tested as single agents, and mixtures of ALA, GLA, LA, and OA approximately simulating typical compositions of dietary oils were also tested. (All UFAs were obtained from Nu-Chek Prep, Elysian, New York.)

In addition, various agents such as epinephrine, acetylsalicylic acid, caffeine and lithium were tested in combination with UFAs for potential synergistic effects using extra available micro-organ cultures, but inconclusive results of these multi-agent tests are not reported.

## Results

**Figure 1:**
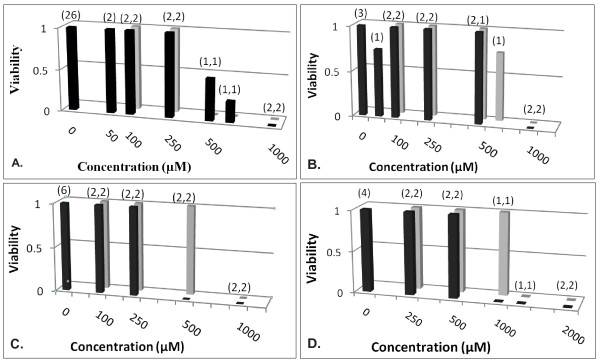
**Cytotoxic activity of UFAs exposed to micro-organs from malignant and normal tissue explants**. **A**: Viability values (0 = sensitive, 1 = resistant) are shown for a metastatic ovarian tumor exposed to GLA (black square) and ALA (Grey square); 26 controls (represented as 0 μM GLA) were all viable. **B**: Results for a primary ovarian tumor. Viability values of 0 at 2 mM (two each for GLA, ALA) are not shown. Four control values were 1.0 (3) and 0.75 (1). **C**: Results for a glioblastoma tumor; 6 control were all viable. **D**: Results for a normal stromal tissue specimen; all 4 controls were viable. A numeric pair (m, n) depicts m and n identical results, respectively, for GLA and ALA represented by the bar below. All four experiments used 10% fetal calf serum.

**Figure 2:**
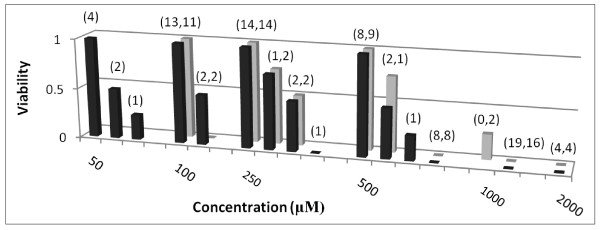
**Aggregate data for UFA exposure to micro-organs from malignant tumor explants in 5-10% fetal calf serum**. Viability values are shown for 153 micro-organ cultures from malignant tumors exposed to GLA (Black square) or ALA (Grey square) in 5% or 10% fetal calf serum. Tumor types tested, as detailed under methods, were ovarian adenocarcinoma, breast carcinoma, pancreatic carcinoma, melanoma, and glioblastoma. Parenthesized values above bars here and in the following two figures represent multiple data points as in Fig. 1. For example, at a 50 μM GLA concentration, the viability readings were 1.0 (resistant) for 4 micro-organ cultures, 0.5 (intermediate) for 2, and 0.25 (intermediate-sensitive) for 1.

**Figure 3:**
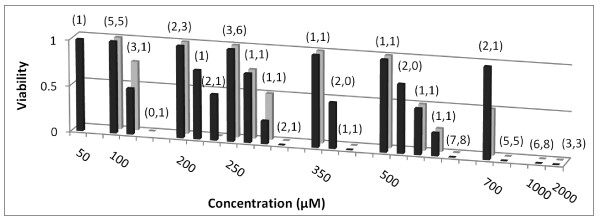
**Aggregate data for UFA exposure to micro-organs from malignant tumor explants in 5-10% human serum**. Viability values are shown for 103 micro-organ cultures from malignant tumors exposed to GLA (Black square) or ALA (Grey square) in 5% or 10% human serum. (Two viability readings of 0 for 2.5 mM GLA are not shown.)

**Figure 4:**
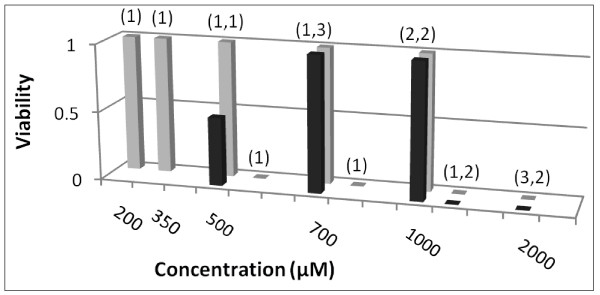
**Aggregate data for UFA exposure to micro-organs from malignant tumor explants in 30-40% human serum**. Viability values are shown for 22 micro-organ cultures from malignant tumors exposed to GLA (Black square) or ALA (Grey square) in 30% or 40% human serum. (Viability readings of 0 for 4 mM GLA and ALA, one each, are not shown.

Figs 1, 2, 3 and 4 show FCA analysis of viability for 282 micro-organ cultures derived from tumors of five malignant types (ovarian adenocarcinoma, breast carcinoma, pancreatic carcinoma, melanoma, and glioblastoma) that were surgically excised from 22 patients and exposed to GLA and ALA at different concentrations. FCA results for normal stromal tissue are also shown in Fig. 1. Additional results for a benign ovarian tumor from another patient, for two other normal stromal tissue specimens, and for tests of other UFAs and UFA mixtures are summarized but not shown.

Fig. 1 shows cytotoxic activity of UFAs exposed to ovarian carcinoma (Fig. 1A, 1B), glioblastoma (Fig. 1C) and normal stromal tissue (Fig. 1D), all cultured in 10% fetal calf serum. Tumor kill is exhibited at a sharp concentration threshold of 500 μM for ALA in Fig. 1A and for GLA in Fig. 1C and at a sharp concentration threshold of 1 mM for GLA and ALA in Fig. 1B, for GLA in Fig. 1A, and for ALA in Fig. 1C. Cytotoxic activity of UFAs exposed to normal stromal tissue shown in Fig. 1D is in about the same concentration range as for malignant tumors. Similar results, not shown, were obtained for two other normal stromal tissue explants and for one benign ovarian tumor. As detailed in the caption for Fig. 1, 39 of 40 control micro-organ cultures in these experiments had viability value 1 (resistant) and one had viability value 0.75.

Figs 2 and 3 show aggregate data for malignant tumors tested in 5-10% fetal calf and in 5-10% human serum, respectively. As discussed in methods, intermediate viability readings in these and Fig. 4, which were assigned values of 0.25, 0.5, and 0.75 for charting purposes and which comprise only 14% of all reported viability values, are less reliable indicators of agent effects *in vivo *than sensitive or resistant readings. While the complete set of viability values is thus not amenable to close data analysis, the significant feature in Figs 2 and 3 is the drop in these values from mostly 1 (resistant) at GLA or ALA concentrations of 250 μM or below to 0 (sensitive) at concentrations of 1 mM and above (with the exception of two viability values of 0.25 for ALA at 1 mM shown in Fig. 2).

Each malignant tumor tested using 5-10% fetal calf or human serum exhibited a sharp cytotoxic concentration threshold between 500 μM and 1 mM for GLA and ALA, varying by explant and agent. With the two exceptions noted above, viability values were 0 at and above this concentration threshold, but were usually 1 below 50% of that concentration.

For experiments conducted in 30% or 40% human serum, cytotoxic activity was consistently observed for GLA and ALA at but not below a 2 mM concentration (Fig. 4). Isolated tests (not shown) for a total of 60 micro-organ cultures from 8 surgical explants using eicosapentaenoic acid (EPA), linoleic acid (LA), and various mixtures of LA, oleic acid (OA), GLA and/or ALA, including ALA, LA and OA in proportions approximately simulating flaxseed oil composition indicate cytotoxic activity consistent with that for GLA and ALA (comparing total molarities of UFAs for mixed agents).

Micro-organ cultures were generally exposed to agents throughout the 7-day duration of the experiment, with a 2-day exposure period used for 33 micro-organ cultures from three malignant specimens and a 5-day exposure period used for 36 micro-organ cultures from two malignant specimens. No study of the relationship between activity and exposure time was performed, but no differences in cytotoxic activity of GLA and ALA based upon varying exposure times were apparent.

## Discussion

It is noteworthy that for each of 22 surgical explants of five malignant types, GLA and ALA each exhibited greater than 90% cytotoxic activity at a sufficiently high concentration threshold. These concentration thresholds for antitumor activity, however, ranging from 500 μM to 2 mM depending upon explant, agent and serum concentration, are much higher than the UFA concentrations of 10 to 100 μM observed to kill tumor in most previously reported *in vitro *studies using tissue culture cells [1,2,5,19].

These FCA findings, however, are consistent with clinical and animal studies in which tumor shrinkage was achieved using localized administration methods [22,27-29,33] providing UFA concentrations as high as 6 mM [33] but not using systemic agent delivery [34-36]. Of particular interest is the parallel to the rat glioma study cited earlier, in which tumor kill but no damage to adjoining normal tissue was observed using 2 mM but not lower concentrations of intratumorally infused GLA [22].

FCA results for normal stromal tissue, on the other hand, support no clear interpretation, since such tissue does not naturally aggregate into micro-organs, has not been previously studied in the FCA assay, and has no record of predictive accuracy for agent effects on normal tissue *in vivo*. Viability values showing cytotoxicity for normal stromal tissue in the same UFA concentration range as for malignant tissue are also inconsistent with *in vitro *[1-3,5,10,12,16-18], animal [22] and clinical [29,33] findings demonstrating full viability of normal tissue at UFA concentrations that killed malignant cells, as high as 2 mM [22] to 6 mM [33]. Nevertheless, these FCA results for normal tissue raise the possibility that the cytotoxic effects observed in all reported FCA tests could represent nonselective cell damage caused by UFA agents at high concentrations.

Alternatively, UFA cytotoxicity to normal tissue could potentially be associated with alterations of the extracellular cell matrix (ECM) caused, for example, by tissue disruption during Tissumizer processing or other assay preparations. The difficulties of simulating the physiological ECM and of maintaining viability for normal cells in culture have been noted [47]. These considerations suggest the hypothesis that fluidization of the plasma membrane caused by UFAs [21,48,49] is a key event triggering ultimate tumor cell death, as has been proposed [50,51].

Malignant cells are characterized by a disorganized ECM [52-56] and supporting cytoskeleton [56-59], in sharp contrast to the well-structured such components of normal cells. Surface bleb formation has been observed beginning as early as 15 minutes after viral transformation to the malignant state [53,56-59]. Some studies indicate that malignant cells have more rigid plasma membranes than normal cells [52] with 2-3 times higher cholesterol content [60], which could perhaps be compensatory for decreased stability of the ECM and cytoskeleton.

Pronounced changes in membrane lipid composition of cultured tumor and normal cells result from UFA supplementation of culture media. Multifold increases in membrane free fatty acid percentages corresponding to added UFAs are observed within hours [61-64]. With UFA added at a 300 μM concentration, the corresponding percentage of total membrane lipids reaches as high as 60% [63]. Membrane lipid changes are 90% complete within about 16 hours [61-64] and persist at least 48-72 hours in unsupplemented culture media [64].

Malignant cell membranes, however, exhibit more pronounced ultimate changes in lipid composition corresponding to ingested UFAs in dietary supplementation studies [60] than do normal cell membranes, and achieve greater fluidization upon exposure to UFAs [21,60]. Supplementation by ALA or oleic acid, in particular, caused fluidization and destabilization in model membranes, an effect related to the hook and boomerang shapes, respectively, of these molecules [65]. Sharp peaks of membrane fluidization were observed in cultured human cancer cell membranes 3 days after exposure to ALA [21] and to linoleic acid [49] with a less pronounced peak for oleic acid at 2-4 days [49].

Apoptosis observed after exposure of malignant cells to UFAs [6,9,14,15,22,66] could thus be triggered by a critical membrane destabilization event resulting from deficiencies in ECM structure and in homeostatic control of membrane fluidity. Tumor cells packed in a tight array could be less vulnerable to membrane destabilization, which would be consistent with the observation that the concentration threshold for significant GLA effect against gliomas varied from 20 μM for monolayer cultures to 200 μM for three dimensional spheroid preparations and 2 mM *in vivo *[66].

If these FCA assay results are predictive of UFA serum levels required for clinical response, as has been the case for other chemotherapeutic agents [42], they present a particular challenge for successful systemic clinical treatment. Albumin sharply attenuates the antitumor activity of UFAs [8,67,68], consistent with FCA assay results noted above showing significantly diminished GLA and ALA activity in 30-40% human serum. Whether intravenous or oral UFA delivery was used, rapid binding of UFA agents to albumin in serum would significantly reduce serum levels of the active unbound form. This constitutes a significant limitation given that under normal conditions, total human serum free fatty acid concentration is about 300 μM [69].

Duration of elevated UFA serum levels also appears to be a significant factor in clinical efficacy, as suggested by time and concentration studies using bladder cancer cell lines with viability measured five days after agent exposure. Tumor kill was achieved with 30-minute exposure to GLA at 1 mM, 1 hour at 500 μM or 2 hours at 125 μM [7], demonstrating a more than linear advantage for application over an extended time frame. This increased cytotoxic effect with longer exposure period is consistent with the time frame of membrane UFA absorption noted earlier, with changes in lipid composition reaching a plateau at about 16 hours and persisting at least 2-3 days.

### A proposed approach for achieving sustained high serum levels of unbound UFAs

One approach for achieving high serum levels of unbound UFAs over an extended time period is to utilize three concurrent physiological perturbations in conjunction with high-dose UFA ingestion: fasting, exercise and caffeine. These and high-dose ingested UFA can each boost serum free fatty acid levels by up to 100%, and combinations can elevate total serum free fatty acid levels into the 1-2 mM range [70-78]. Flaxseed oil, a triglyceride blend consisting of about 58% ALA, 19% oleic acid, 14% linoleic acid and 9% saturated fatty acids, would provide a safe dietary UFA source. This oil, with its high ratio of ω-3 to ω-6 components, would not be subject to risks of tumor growth promotion at suboptimal levels as noted earlier for ω-6 agents [15,20-23].

Specifically, these combined elements could be provided through a regimen consisting of an overnight fast and then ingestion of flaxseed oil without added vitamin E or other antioxidants at 3.33 grams per kilogram of body weight between 9-10 AM, followed by 200 mg caffeine dosages at 10 AM and again at 11 AM. Beginning at 11 AM, the subject would exercise for 90 total minutes, with intermittent short rest periods as needed, at moderate intensity, e.g., at 55% of VO2max or 70% of maximum heart rate, within the limits of the subject's capability. The fast would then terminate at 6 PM. This schedule would be repeated over three consecutive days. Given unknown risks, this regimen would be suitable only for application in a clinical study involving subjects having no proven treatment options, with daily complete blood counts conducted and other precautions taken.

Combined fasting, exercise and caffeine in untrained women per specifications similar to the above, but without fat ingestion, resulted in serum free fatty acid levels averaging 1.9 mM [73]. Increased free fatty acid serum levels achieved under such conditions persisted for several hours with continued fasting [75,77]. It thus appears that total serum free fatty acid levels of at least 1.5 mM could be obtained between 1 and 6 pm each day of this proposed regimen, with about half of serum fatty acids consisting of UFAs from flaxseed oil, as can be roughly projected based on oral ingestion studies of 30-40 grams [79] or 100 grams [71] of fats. This would yield a concentration of at least 750 μM for unbound UFAs in serum during a total period of 15 total hours over three consecutive days. Note that this figure of 750 μM is appropriately compared to the UFA tumor kill concentration threshold of 500 μM to 1 mM for FCA tests using low serum concentrations, since unbound UFA concentrations in FCA tests using high serum concentrations would be significantly diminished by albumin binding.

Several physiological potentiating factors could enhance clinical efficacy. Lipid peroxidation, which has been found to sharply promote UFA antitumor activity [1-5,11,16,19,46,80-82], could be triggered clinically by reactive oxygen and nitrogen species released during immune surveillance of tumor cells [83]. Changes in plasma membrane shape during mitosis could yield additional vulnerability to UFA-induced membrane destabilization for tumor cells in that phase of the cell cycle. This could in turn result in propagating death in neighboring cells through a cascade of released lipid peroxidation products.

Elevated local concentrations of free fatty acids would occur in tumors as a consequence of increased secretion of lipoprotein lipase [84], which may be caused by increased metabolic requirements of malignant cells and may thus be more pronounced under fasting conditions. In addition, plasma membrane lipid turnover would be increased by caffeine at moderate dosages [85,86], while exercise and caffeine would yield an increased ratio of unsaturated to saturated fatty acid levels in serum [75]. Lymphatic transport of ingested lipids may yield increased levels of UFAs in the intestine or peritoneal cavity, which would provide an advantage against tumors in those anatomical regions.

Finally, additional possibilities for clinical benefits are raised by findings that multidrug resistant (MDR) malignant cell lines were as sensitive [7,14,87] or more sensitive [88] to UFA agents than their non-MDR counterparts and that drug resistance could be reversed by UFA application [51,89,90]. This suggests the possibility that should the proposed 3-day combination regimen fail to achieve response, it could still potentially reverse drug resistance in MDR tumor cells or selectively target them and allow response to be achieved in subsequent chemotherapy treatments.

A preliminary test of feasibility of the proposed combination of high-dose flaxseed oil, fasting, exercise and caffeine was conducted for one day only in 2006 by an ovarian cancer patient whose tumor markers had begun to progress during a second chemotherapy regimen. Four months after the last treatment in this regimen, after being warned of unknown risks of this dietary intervention, the patient began an overnight fast and the next morning ingested 225 grams of flaxseed oil plus 100 mg caffeine followed by light exercise and hourly intake of caffeine in a total additional dose of 800 mg over a 6-hour period, after which the fast was terminated.

The patient was able to ingest the flaxseed oil without difficulty, with palatability improved by being blended with a few tablespoons of non-fat yogurt, sweetener and ice cubes to a flowing consistency. A transient drop in CA125 tumor marker level followed this treatment (CA125 values for 54, 28 and 5 days before treatment and then 8 and 28 days after treatment were, respectively, 31, 55, 77 and then 40 and 66). Two subsequent repetitions of this dietary intervention without full adherence to fasting caused no reduction in steadily increasing tumor marker values. The patient subsequently responded to a different chemotherapy regimen and survived two additional years.

Although burdensome for patients and subject to significant individual variations in unbound UFA serum levels achieved, the proposed 3-day regimen can provide a mechanism to initially test whether UFAs at high unbound serum levels over an extended period could yield clinical response. Stage III ovarian cancer patients with recurrent or multi-drug resistant disease, as indicated by rising CA-125 tumor marker values, would be ideal subjects for a clinical trial given their typically high performance status, lack of proven curative options, location of tumor in the peritoneal cavity, and ability to track response through monthly CA-125 blood test results.

High serum concentrations of unbound UFAs can also be achieved using combined intravenous administration of a lipid emulsion and heparin. Studies using Intralipid and heparin administered intravenously to normal volunteers yielded total serum free fatty acid levels of 1.3 mM [91] and 1.67 mM [92] respectively 3 and 5 hours after initiation of infusion. Also, a custom UFA blend could prove more effective than flaxseed oil, for example, with GLA and EPA substituted for linoleic acid and saturated fatty acid components. For initial testing, however, the proposed combination of oral flaxseed oil, fasting, exercise and caffeine would appear to offer less risk of unknown adverse effects and also the potentiating factors cited above.

Should sustained high serum levels of unbound UFAs achieved through some such approach yield major response, it would be of particular interest to explore whether aberrant properties of the malignant plasma membrane and ECM that are in some cases independent of genomic instability [93] could underlie such effects. It has been demonstrated, for example, that one dose of ionizing radiation to nonmalignant human mammary epithelial cells causes heritable loss of organization, aberrant cell-to-ECM interactions and loss of tissue specific architecture in progeny of irradiated cells, all characteristic of neoplastic progression, but without the occurrence of genetic changes [94].

In a series of studies, Clarence Cone [95,96] demonstrated differences in transmembrane voltage and associated ionic concentrations between normal and malignant cells and was able to influence mitotic behavior by experimental manipulation of these environmental factors. Nuclei of malignant cells transplanted into normal oocytes in frogs or mice have yielded, respectively, fully functional tadpoles [97] or post-implantation mouse embryos with high [98] or mixed [99] degrees of tissue development. Many studies that illuminate non-genomic, membrane-related characteristics of malignancy have been comprehensively reviewed [100].

## Conclusions

A sharp, high tumor kill concentration threshold for GLA, ALA, and other UFAs was determined using fresh human tumor explants analyzed with the clinically predictive FCA assay system. This concentration threshold of 500 μM to 2 mM for antitumor activity, depending upon explant, agent and serum concentration, is consistent with *in vivo *and clinical results and indicates a need for a dose-intensive approach in the application of UFAs to cancer treatment.

An oral clinical regimen is proposed that could push the area under the curve for serum concentration of unbound UFAs over time to much higher levels than previously achieved and into the range that could yield clinical response. If safety is demonstrated and if response is achieved for some patients, efficacy could potentially be achieved more consistently and patient burden diminished with intravenous administration of a UFA emulsion and heparin, as noted above.

## Competing interests

The author declares that they have no competing interests.
